# Antibiotics and multi-resistant organisms

**DOI:** 10.1186/1824-7288-41-S1-A45

**Published:** 2015-09-24

**Authors:** Chryssoula Tzialla, Elisa Civardi, Margherita Pozzi, Mauro Stronati

**Affiliations:** 1 Neonatal Intensive Care Unit, Fondazione IRRCS Policlinico San Matteo, Pavia, Italy

## 

The development of antibiotic resistance is a normal evolutionary process for microorganisms, but it is accelerated by the selective pressure exerted by the widespread use of antibacterial drugs. 

Nearly all very low birth weight infants admitted to neonatal intensive care units (NICUs) receive an empirical antibiotic treatment in the first days of life, despite the evidence of sterile cultures [1,2]. 

Several studies show that widespread and inappropriate use of antibiotics is very common in NICUs and that the overuse of antibiotics like third-generation cephalosporins favors the emergence of multi-resistant (MR)bacteria in NICUs [1,2]. A recent study describes1,106 episodesof bacteremiain a NICU: 35.5% were caused bygram-negative bacteria (GNB), and18.6%byMR strains. In the multivariate analysisthe authors identifiesasindependent risk factorforthe emergence of resistance the exposure to third-generation cephalosporins, and carbapenems[3].

Glycopeptide antibiotics remain adequate as treatment of most staphylococcal infections and vancomycin is the drug of choice for serious infections [1,4]. However in case of infections due to Gram-positive bacteria (GPB) unresponsive to vancomycin, linezolid has been the most used in neonatal settings. Several novel antibiotics active against GPB are currently in diverse phases of development [1,4].

GNB are often resistant to at least one class of antibiotics that is used as standard, and may be from time to time resistant to all-first-line antibiotics. The development of new antibiotics active against resistant GNB has not progressed in parallel with increasing rates of resistance. This scenario of limited therapeutic options has prompted renewed interest in older and more toxic antimicrobials [4,5].

Since antibiotic resistance cannot be eradicated, different strategies have been proposed to slow the development and spread of MR that aim to [6,7]: 1)improve the knowledge of MR bacteria and antibiotic use through surveillance at national and international levels,2) conserve the effectiveness of existing treatments and3) stimulate the development of new antibiotics, alternativetreatmentsand preventive strategies

Antibiotic resistance is a public health concern worldwide. Although some important molecules are currently in diverse phases of development for treatment of infections due to resistant GPB, very few drugs have been reached advanced stages of development for infection due to MR gram-negative bacilli. At this point it is essential to preserve the efficacy of existing drugs while efforts to develop new treatment options proceed.
